# SPINE: SParse eIgengene NEtwork Linking Gene Expression Clusters in *Dehalococcoides mccartyi* to Perturbations in Experimental Conditions

**DOI:** 10.1371/journal.pone.0118404

**Published:** 2015-02-25

**Authors:** Cresten B. Mansfeldt, Benjamin A. Logsdon, Garrett E. Debs, Ruth E. Richardson

**Affiliations:** 1 Department of Civil and Environmental Engineering, Cornell University, Ithaca, NY, United States of America; 2 Sage Bionetworks, Seattle, WA, United States of America; 3 Department of Biological and Environmental Engineering, Cornell University, Ithaca, NY, United States of America; National Centre for Cell Science, INDIA

## Abstract

We present a statistical model designed to identify the effect of experimental perturbations on the aggregate behavior of the transcriptome expressed by the bacterium *Dehalococcoides mccartyi* strain 195. Strains of *Dehalococcoides* are used in sub-surface bioremediation applications because they organohalorespire tetrachloroethene and trichloroethene (common chlorinated solvents that contaminate the environment) to non-toxic ethene. However, the biochemical mechanism of this process remains incompletely described. Additionally, the response of *Dehalococcoides* to stress-inducing conditions that may be encountered at field-sites is not well understood. The constructed statistical model captured the aggregate behavior of gene expression phenotypes by modeling the distinct eigengenes of 100 transcript clusters, determining stable relationships among these clusters of gene transcripts with a sparse network-inference algorithm, and directly modeling the effect of changes in experimental conditions by constructing networks conditioned on the experimental state. Based on the model predictions, we discovered new response mechanisms for DMC, notably when the bacterium is exposed to solvent toxicity. The network identified a cluster containing thirteen gene transcripts directly connected to the solvent toxicity condition. Transcripts in this cluster include an iron-dependent regulator (DET0096-97) and a methylglyoxal synthase (DET0137). To validate these predictions, additional experiments were performed. Continuously fed cultures were exposed to saturating levels of tetrachloethene, thereby causing solvent toxicity, and transcripts that were predicted to be linked to solvent toxicity were monitored by quantitative reverse-transcription polymerase chain reaction. Twelve hours after being shocked with saturating levels of tetrachloroethene, the control transcripts (encoding for a key hydrogenase and the 16S rRNA) did not significantly change. By contrast, transcripts for DET0137 and DET0097 displayed a 46.8±11.5 and 14.6±9.3 fold up-regulation, respectively, supporting the model. This is the first study to identify transcripts in *Dehalococcoides* that potentially respond to tetrachloroethene solvent-toxicity conditions that may be encountered near contamination source zones in sub-surface environments.

## Introduction

Strains of *Dhc* are primarily studied for their ability to respire and biotransform haloorganics into non-toxic end products [1–3]. This anaerobic bacterium oxidizes diatomic hydrogen and reduces haloorganic compounds (e.g., chloroethenes, chlorobenzenes, chlorophenols, polychlorinated biphenyls, and dioxins) [2]. These haloorganics include contaminants that are carcinogenic, recalcitrant, and widely distributed in hazardous-waste sites. *Dhc* is an attractive organism to use in bioremediation and has been increasingly employed at field sites contaminated with PCE and TCE (tetrachloroethene and trichloroethene, respectively) to convert these chlorinated solvents into non-toxic ethene [3, 4].

However, the mechanisms of organohalorespiration and stress response in *Dhc* are not well characterized. Previous studies have identified the terminal enzymes reducing the organohalide as reductive dehalogenases (RDases) [5, 6]. Classical molecular techniques, such as over-expressing or knocking-out target genes, have as of yet proven elusive because of the non-standard physiology of the organism [7]. As a consequence, only select RDases and few other enzymes have been directly characterized in *Dhc* [6, 8–10].

To gain an understanding of the organization, operation, and regulation of key metabolic processes and stress-responses of the organism, large datasets are created using high-throughput transcriptomic and proteomic methods. Previous studies applied this methodology to infer specific properties of *Dhc* such as the organization of the central carbon pathway [11] and the nitrogen fixation potential [12]. These studies, in addition to providing evidence for specific hypotheses, developed large datasets available for meta-analyses to determine global trends. In general, applying computational methods to reanalyze previously generated data provides a method to detect relationships and hypotheses that were previously unidentified.

This study focuses on the results of the high-throughput study previously described by Mansfeldt et al. [13]. The resulting dataset contained multiple qualitative and quantitative perturbations of experimental conditions experienced by *Dhc*, genome-wide transcriptomic response measurements, and ample evidence that the coordinated changes in gene expression were a function of the state of *Dhc* (i.e., the mRNA pools reached a steady state following continuous exposure to particular conditions). We designed a statistical model to leverage these features to identify strong associations between experimental conditions and coordinated changes in gene expression.

Previous studies have yet to combine all of the methodological pieces necessary to identify conditional statistical associations between gene expression clusters and experimental conditions in data with more variables than observations. Whereas multiple authors propose constructing sparse statistical networks, [14–17], these studies do not consider clustering variables to either account for modular expression of genes or enforce dimensionality reduction, and will therefore suffer from a high multiple-hypotheses testing burden. Alternatively, methods that do perform clustering either do not allow for conditional relationships to remove non-specific associations [18, 19] or use penalized objective functions with penalties such as lasso with poor type-I error-control properties [20]. The SParse eIgengene NEtwork (SPINE) method with the vbsr technique provides an alternative that addresses all of these challenges specifically for high dimensional data-sets.

We infer a SPINE that directly captures the flow of information from the experimental conditions through clusters of gene transcripts that are tightly co-expressed within the data. To identify the effect of experimental perturbations on the transcriptome of *Dhc*, the SPINE method combines two data-dimensionality reduction techniques (clustering and sparse conditional dependence models) in a new way. We first clustered the expression of 1,419 genes from *Dhc* (that were monitored on a microarray) into 100 clusters based on their patterns of gene expression across 47 experimental conditions as defined by 42 experimental condition variables. These experimental conditions include various parameters such as the respiration rate, the electron acceptor type, and the duration of the experiment; a full list of these conditions are defined and detailed in S1 File. This transcript-clustering step reduces the model dimensionality and increases the statistical power to identify associations between experimental conditions experienced and the transcriptome expressed by the organism by reducing the multiple hypothesis testing burden. This process is similar to other popular methods such as the WGCNA and eigengene network analysis [18, 19]. A cluster is defined as a set of gene transcripts that coordinately respond in the experimental dataset. We summarize the gene expression of each cluster by extracting the principal axis of variation (eigengene) across the experimental dataset to characterize the aggregate expression of members of the cluster using a standard approach (principal component analysis; PCA). This is similar to the method of Langfelder and Horvarth [19].

Even after the data dimensionality reduction from clustering, numerous non-specific statistical associations between experimental conditions and gene expression remain. For example, when a given experimental condition activates a cascade of downstream gene-expression clusters, all these clusters will be correlated with that experimental condition. Our method is designed to filter out the majority of these correlations, except for the correlation between the specific experimental condition and the cluster containing gene transcripts directly responding to this condition. Our method excels at filtering the other non-specific false positives. To build the model, we use a sparse regression technique (variational Bayes spike regression; vbsr) that is designed to identify strong statistical relationships between the experimental conditions and clusters when the number of samples (47) is less than the number of variables considered (142). The vbsr technique in the SPINE method approximates the best subset selection and has numerous statistical advantages over alternative approaches (e.g., lasso [21]), including better Type-I error control (i.e., correctly rejecting true negatives) [22]. We use this approach to identify clusters that are directly perturbed by experimental conditions.

The clustering and sparse network inference methodology outlined above was applied to the previously described *Dhc* microarray dataset [13]. In brief, the dataset consisted of ratios of intensity values from Agilent two-color 8x15k complimentary DNA (cDNA) microarrays, chlorinated ethene concentrations, other metabolite concentrations, culture respiration rates, feed rates, types of substrates used, and qualitative descriptions of the state of the culture (e.g., starved, exposed to stressors) resulting from 47 continuously-fed cultures. By monitoring continuously fed cultures, rather than batch fed cultures, the measured and stable values of the RNA pool can be correlated to the experimental conditions (e.g., feed rates, substrate type). The combination of the microarray values with the experimental conditions allows for the direct correlation of the transcript expression with the phenotypic characteristics of the culture.

The model predicted several gene-expression clusters to be strongly associated with experimental conditions experienced by *Dhc*. To begin to validate the constructed model and to investigate a proposed stress response mechanism, follow-up continuously-fed experiments employing quantitative reverse transcription PCR (qRT-PCR) were performed on cultures exposed to toxic (saturating) levels of PCE.

## Results and Discussion

### Gene-expression aggregation

After processing and normalizing the 1,419 transcripts monitored on the microarray, we identified 100 distinct aggregations of gene expression phenotypes using K-means clustering [23]. The experimental data was clustered into 100 distinct groups to balance shrinking the dataset with removing true biological signals. Whereas the optimal choice of the number of clusters is a notoriously ill-posed problem, we experimented with a range of values, from 30–200 clusters. In these experiments (data not shown) we found that for fewer clusters, the within-cluster correlation was much lower and predicted functionally divergent genes were often grouped together. We were concerned we were aggregating gene expression too harshly and were therefore losing important information. Alternatively, for a higher number of clusters, numerous clusters only had a few members and more clusters were highly mutually correlated. In this case, we were concerned we were not aggregating variables as effectively as possible. The choice of a 100 clusters was a good trade-off between these two extremes.

As discussed in the methods, the principal eigengene from each gene expression cluster (*C*1,…, *C*100) was computed and used in all downstream analyses. This eigengene served as a summary of the principal pattern of expression of all the transcripts assigned to that particular cluster. Whereas the principal eigengene is closely related to the centroid of each cluster, we note that the use of eigengenes is more general than using a centroid since additional eigengenes could be selected and incorporated as variables within the network. The direct comparisons between the eigengenes and centroids was beyond the scope of this paper.

Supplementary S1 Table contains the gene assignments for each cluster based on K-means clustering. The median number of members assigned to each cluster was 12 with a standard deviation of 8.04. The minimum and maximum number of members per cluster was four (*C*47) and 44 (*C*91). As a final diagnostic, we also inspect the *R* ^2^ distribution for a given cluster in terms of how much variation the eigengene explains for each individual transcript in that cluster. The clusters are coherent with an overall average *R* ^2^ of 0.73.

**Fig 1:**
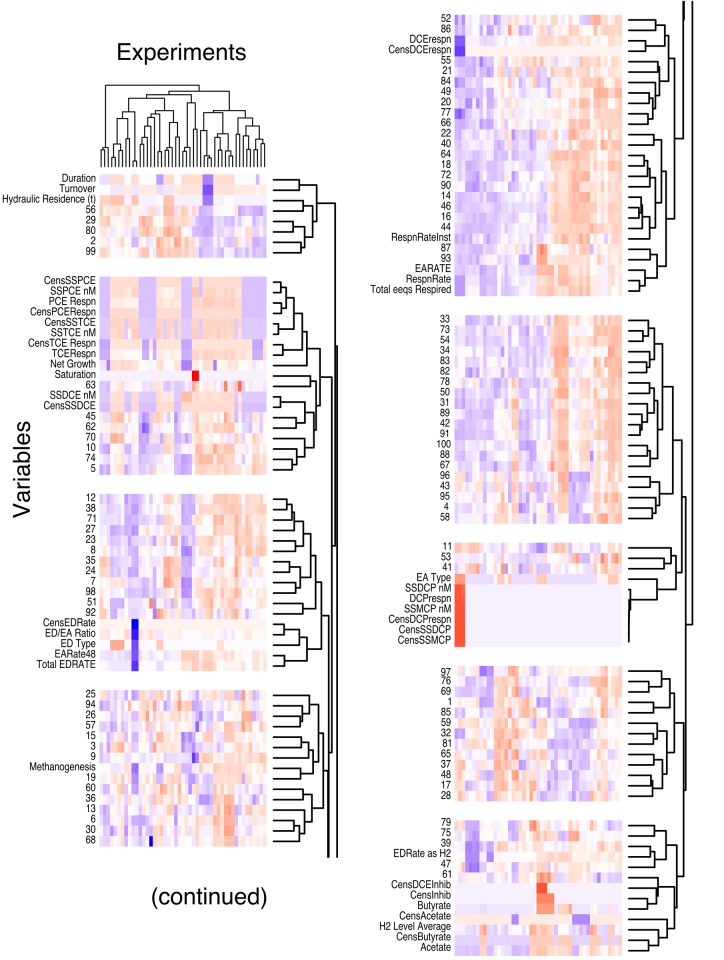
Heatmap constructed for the 100 eigengenes of the clusters and the 42 experimental variables recorded for *Dhc* across 47 experiments with varying conditions. The heatmap is organized by the hierarchal clustering of both the variables (y-axis) and experiments (x-axis). Blue, white, and red indicate a negative, neutral, or positive microarray expression ratio (or low, mid, or high values for experimental variables), respectively. The variables are segregated into distinct blocks by introducing a white space between groups of variables that had distances greater than 0.9 × (maximum hierarchical distance).

Fig. 1 displays a heatmap with a nearest neighbor dendrogram of the 100 eigengenes describing each cluster along with the distinct experimental conditions experienced by *Dhc*. This figure is presented to provide a broad overview of the data modeled in the SPINE. The individual values are transformed to range from negative one (non-abundant or absent; blue) to one (abundant or present; red). Along the x-axis, the majority of the experiments cluster according to biological replicates, as a previous publication noted [13]. Along the y-axis, four groups with five or more transcripts of similarly expressed genes are highly related to multiple experimental conditions experienced by *Dhc*; however, substantial heterogeneity among the individual clusters was present within each of these four groups. Notably, similarly described experimental conditions (e.g., RespnRate and EARate) clustered closely together. In Fig. 1, the heatmap depicts the high level organization of eigengenes into clusters and how these clusters vary as a function of experiment. Overall, this approach successfully displays the global patterns in the dataset; however, this approach displays difficulty in predicting sparse and testable relationships.

### SPINE

To discover relationships among these clusters and noted experimental conditions experienced by *Dhc* and filter the numerous false positives inherit in other methods such as hierarchical clustering, we inferred a SPINE given the experimental conditions. Whereas a functional annotation (e.g., gene ontology enrichment) potentially could establish an optimal number of clusters in other organisms, in *Dhc*, a paucity of functional annotation is available for the identified genes. This lack of information complicates effectively identifying cluster definitions. In addition, coexpression patterns among genes in a gene ontology analysis will be determined primarily by the set of perturbations, which in many cases may be unrelated to known pathway structures. Therefore, an alternative strategy is available by generating SPINE models for a range of cluster sizes and then interrogating the ensemble of models for properties of interest.

Additionally, alternative approaches such as WGCNA were applied to the data, and other approaches such as ARACNE [14] could be applied to the data; these competing methods are not designed to answer the identical questions as the SPINE. Specifically, the SPINE identifies strong conditional dependence relationships among gene expression clusters, whereas WGCNA identifies strong marginal dependencies among gene expression clusters. ARACNE can identify weak conditional dependence relationships, but is inadequate at filtering relationships (e.g., SPINE has a stronger sparsity assumption on the underlying network topology). Recent work suggests that the sparsity assumptions underlying the VBSR model used in SPINE are highly relevant to underlying biological processes [24]. Similarly, community detection approaches such as Infomap [25] could be used on the inferred network, but this approach is unnecessary given the modest size of the network.

**Fig 2:**
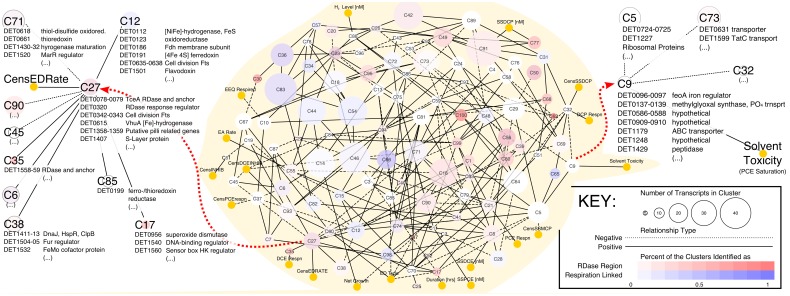
The SPINE (SParse eIgengene Network) inferred for the *Dhc* transcriptomic data (center gray area). Experimental variables are represented as yellow nodes with a consistent size. The size of a transcript cluster node is a function of the number of transcripts in that cluster, and these transcript cluster nodes are colored as either white to red or white to blue. The nodes are colored based on the proportion of their constituent transcripts that are RDase-related (with white and red indicating a lower and higher proportion, respectively) or the proportion of their constituent transcripts identified as other oxidoreductases putatively involved in electron transport (with white and blue indicating a lower and higher proportion, respectively). Both filters are considered simultaneously, allowing purple nodes. The network was visualized in Cytoscape v. 3.0. Red arrows highlight a zoomed in neighborhood view of two clusters that are discussed in the text: (left) members of and neighbors to the *C*27 eigengene, the cluster containing the most highly-expressed RDase *tceA* (DET0079); and (right) members of and neighbors to the *C*9 eigengene, the only cluster connected to the solvent toxicity (saturation) experimental condition in the model.

Fig. 2 presents the network in graphical form. The resulting model consists of a single connected network with 118 nodes that have an average of 4.37 neighbors (connecting edges); 24 variables (clusters or conditions) displayed no connections. This figure depicts the predicted associations between experimental conditions influencing *Dhc* and the gene expression clusters, in which the clusters are colored based on the proportion of genes that are identified as surrounding or encoding key proposed respiratory genes. These genes include reductive dehalogenases (red; includes catalytic subunits, anchoring subunits, and putative regulators) and other non-RDase oxidoreductases putatively identified as involved in respiration (blue; assigned by Seshadri et al. 2005). The darker the node, the greater the fraction of the total members of a given cluster are assigned to each category. In this network, the experimental conditions (represented as yellow nodes) have an average of 1.50 connections per experimental variable, substantially lower than the network average. This suggests that the gene transcripts that are a member of the connecting eigengenes may play key roles in mediating the transcriptomic response to that particular experimental condition.

One of the intensively studied aspects of *Dhc* is the expression pattern of key RDases (*Dhc* strain 195 contains 17 distinct RDase operons). Initially focusing on the behavior of RDases in the network allows a first insight into whether the SPINE model predicted biologically supported interactions. Overall, the SPINE reaffirms the general bifurcation of RDase components that has been previously noted [13, 26]. As shown in Fig. 2, *C*100 is one of the darkest red nodes because eight out of its eleven members are RDase related. These members include the RDase catalytic subunits DET0173, DET1535, and DET1538. The *C*100 cluster is connected to the *C*80 cluster; members of the *C*80 cluster include the DET0180 and DET1545 RDase catalytic subunits. This connection is expected because the expression of these five RDases have previously been shown to display an inverse trend to the overall respiration rate [26–28]. These transcripts are highly expressed under slow respiration rates or starvation conditions.

A notable absence from the RDase list presented above is the dominant RDase: *tceA* (DET0079) [6]. Because this is the major enzyme that has been biochemically shown to pass electrons to trichloroethene (TCE), dichloroethene (DCE), and vinyl-chloride (VC) to produce ethene (ETH), DET0079 was anticipated to not interact with the low-respiration linked RDases. The *tceA* transcript clusters in the *C*27 eigengene (the central node of Fig. 2, left). Other notable members in this *C*27 cluster include the *tceA* membrane anchoring protein (DET0078), a predicted regulatory protein of *pceA* (DET0320; *pceA* is a PCE-to-TCE dechlorinating RDase), the putative S-layer cell-wall protein (DET1407), a subunit of the cytoplasmic [Fe]-hydrogenase Vhu (DET0615), and members of the Fts cell division complex (DET00342-0343). Additionally, *C*27 is connected to *C*80 and *C*100 through *C*12 and *C*85, respectively. *C*12 contains numerous respiration linked transcripts (Fig. 2; discussed below), and *C*85 contains a transcript encoding for the putative ferrodoxin/thioredoxin reductase. Therefore, the connection of both the fast- and slow-respiration linked RDases (i.e., the RDases in *C*27 and *C*80/*C*100, respectively) to identical clusters containing transcripts encoding respiration linked enzymes supports the involvement of RDases in respiratory processes.

Additionally, the *C*27 cluster is directly connected to *C*6, *C*12, *C*17, *C*35, *C*38, *C*45, *C*71, *C*85, *C*90, and CensEDRate (Fig. 2, left). The CensEDRate is a variable that is either one or zero depending on whether an electron donor was or was not provided, respectively. As can be seen from the heatmap in Fig. 1, the majority (45 out of 47) of experiments analyzed in this dataset were provided an electron donor; the *tceA* transcript displayed an average ratio of 17.8±4.5 for these samples. For the two experiments that did not receive an electron donor, *tceA* displayed an average ratio of 0.66±0.68. Therefore, the connection between *tceA* and the CensEDRate emphasizes the fact that when the electron donor was not provided in the dataset, the abundance of *tceA* was low.

Of the clusters that are connected to *C*27 (Fig. 2, left), only the *C*35 cluster contains another RDase (DET1559). This RDase has been previously detected during growth in studies monitoring the RNA or protein levels [29–31]. *C*27 also maintains an important positive relationship to *C*12. The *C*12 cluster contains transcripts for cell division proteins (DET0635-0638) and several non-RDase oxidoreductases predicted to be involved in respiration including the following: the [NiFe]-hydrogenase FeS binding subunit (DET0112), a formate dehydrogenase like (Fdh-like) membrane subunit (DET0186), and a member of the NADH-ubiquinone oxidoreductase (DET0923). *C*12 is also directly connected to the *C*66 cluster. The *C*66 cluster captured the majority of the remaining respiration linked transcripts. Representative members in this cluster include transcripts for the Hup [NiFe]-hydrogenase subunits (DET0110-0111), the Hym [Fe]-hydrogenase (DET0145-0148), a Fdh-like oxidoreductase (DET0187), and a member of the NADH-ubiquinone oxidoreductase (DET0924).

One of the other notable interactions the model describes is the connection of *C*9 to the solvent toxicity experimental variable (Fig. 2, right). Notably, *C*9 is not near the variable for the saturated levels of PCE in the simple hierarchical clustering technique presented in Fig. 1. However, members of the *C*9 cluster are not expressed for the majority of conditions and are only detected in high abundance under the specific stress conditions of saturated levels of PCE that induce solvent toxicity. Two transcripts in this cluster, DET0137 and DET0097, maintain annotations potentially related to sensing and responding to stress (as a methylglyoxal synthase and DtxR iron-based regulator, respectively). To determine whether this was a prediction entirely specific to SPINE, we also built a network using the weighted graph correlation network analysis (WGCNA) [18, 19]. Whereas overlap was noted between the two approaches in terms of genes predicted to be linked to saturation (DET0137, DET0586, DET0587, DET0588, and DET1179), SPINE uniquely predicted the iron regulator DET0097, among others (DET0096, DET0138, DET0139, DET0909, DET0910, DET1248, and DET1429), to be connected to solvent toxicity. Because the majority of these additional transcripts maintained a hypothetical annotation, only DET0137 and DET0097 are explored in more detail as potential biomarkers of solvent toxicity.

### Validation of the stress response to solvent toxicity in *Dhc*

**Fig 3:**
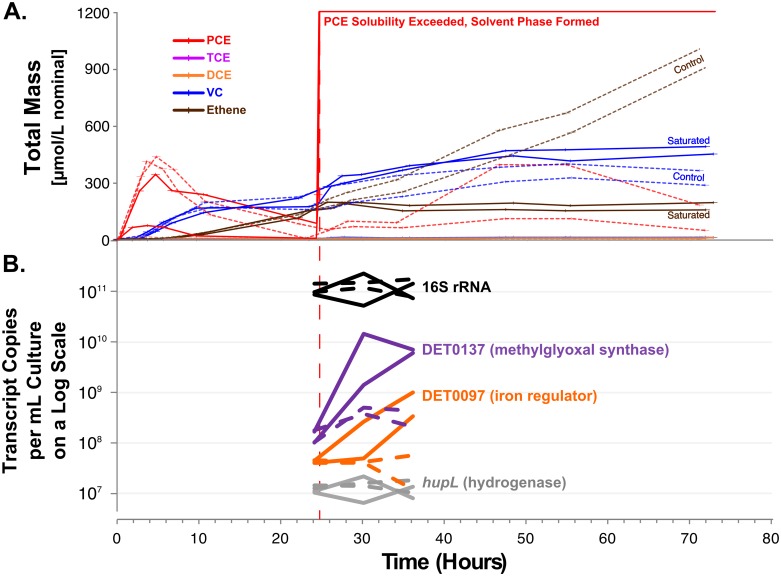
The response of selected *Dhc* strain 195 transcripts to solvent toxicity. (a) A line plot of the concentrations of PCE, TCE, DCE, VC, and ETH during the continuously fed experiment for the saturated (solid lines) and control (dashed lines) cultures. The biological duplicates are presented individually. Excess PCE was spiked into the experimental cultures at 24 hours. TCE and DCE levels were always below 5 µmol/L. (b) A line-plot of the transcript copies per mL of culture presented on a log scale for DET0097 (a putative regulator), DET0110 (*hup* hydrogenase large subunit), DET0137 (a putative methylglyoxal synthase), and 16S rRNA. Data for the saturated and control cultures are represented by solid and dashed lines, respectively.

Given the strong linkage between solvent toxicity and the *C*9 cluster, we looked to validate this model-predicted relationship. To biologically validate this relationship, a continuously fed experiment was performed. When saturating levels of PCE occur, *Dhc* is known to experience solvent toxicity. Four cultures were allowed to equilibrate to the continuous feeding conditions at moderate rates for 24 hours (Fig. 3). After 24 hours, two of the four cultures (A and B) were spiked with 140 *μ*L/L (1.37 mM nominal) of pure PCE, well above its solubility limit. By exceeding the solubility limit, a free phase of PCE solvent formed in the cultures. This injection of PCE immediately halted the production of ethene; after approximately 24 hours, reductive dechlorination then ceased entirely (Fig. 3a). The two control cultures that did not receive a spike retained a high-rate of reductive dechlorination, accumulating ethene as the final product.

The qRTPCR results for the DET0097 and DET0137 transcripts selected from the *C*9 cluster are presented in Fig. 3B alongside results for the comparative controls of the hydrogenase *hupL* DET0110 (a highly expressed member of *C*66, connected to high respiring conditions [32]) and 16S rRNA (a general indicator of the abundance of *Dhc* cells). After twelve hours of exposure to the solvent toxicity conditions, DET0137 was 46.8±11.5 fold up-regulated (from the initial pre-spiked culture) in the cultures experiencing solvent toxicity compared to 2.6±2.0 for the control cultures; DET0097 was on average 14.6±9.3 fold up-regulated in the cultures experiencing solvent toxicity compared to 0.8±0.7 in the control cultures. In comparison, the DET0110 and 16S rRNA transcripts did not display significant changes in expression levels.

The follow-up study finds that DET0137 and DET0097 do respond to the solvent toxicity state, and these two transcripts increase in abundance, suggesting a positive relationship. Therefore, this follow-up study reinforces the SPINE predictions.

The up-regulation of the putative methylglyoxal synthase and the putative iron regulator provide hypotheses for the mechanism of the solvent stress response in *Dhc*. DET0097 is predicted to be a helix-turn-helix (HTH) transcriptional regulator of the DtxR class. This class of regulators have been shown to be involved in the regulation of iron metabolism [33]. Additionally, iron-regulating proteins have been shown to be involved in the regulation of the oxidative stress pathway across a wide distribution of bacteria [34]. DET0097 is also notable in that it is unique to *Dhc* strain 195 in the *Dehalococcoides* genus. Therefore, although DET0097 is a good specific biomarker to monitor *Dhc* strain 195 for this stress, this result cannot be generalized to other strains. However, the methylglyoxal synthase (DET0137) is conserved across all *Dhc* strains and is a promising candidate for diagnosing stress in field-site *Dhc* populations. The methylglyoxal synthase is unlikely to act on PCE directly because this enzyme is predicted to produce methylglyoxal.

However, a notable feature of methylglyoxal is its toxicity. Previous studies have found that low amounts of methylglyoxal can halt cellular growth in a wide range of organisms with the most detailed results available for *E. coli* [35]; this halting of growth is one of the proposed roles of methylglyoxal in the cell. Additionally, the methylglyoxal synthase has been previously shown to be responsive to phosphate limitation and imbalances in the rate of glycolysis with carbon uptake [36]. Therefore, the SPINE analysis may have identified a crucial control mechanism of the cell: this methylglyoxal synthase will be turned on under solvent toxicity conditions to arrest cellular growth. Previous studies on *Dhc* have found that a similar but distinct methylglyoxal synthase (DET1576) was up-regulated under nitrogen limitation conditions [12]. Therefore, the expression of this gene or genes of this type may act as a useful biomarker for extreme stress conditions in which cells halt their metabolism.

Overall, the design and implementation of a SPINE model allowed us to consider a dataset in which more variables (142 after transcript clustering) were present than observations (n = 47) for *Dhc*. The model predicted two transcripts, DET0137 (the putative methylglyoxal synthase) and DET0097 (the putative iron regulator), to be directly connected to the solvent toxicity condition; these relationships were confirmed in a follow-up study. Additionally, these transcripts may serve as useful field site biomarkers. Therefore, this network inference method may be useful for analyzing datasets covering other organisms or applications to identify strongly supported biological responses to experimental conditions.

## Materials and Methods

### Microarray data processing and transcript clustering

The microarray data used to construct the SPINE resulted from the scan of an Agilent two-color 8 × 15k oligonucleotide array that captured Cy3 and Cy5 labeled reverse-transcribed complimentary DNA (cDNA) (Agilent Technologies, Santa Clara, CA). The individual experimental results were run in the Cy5 (red) channel. A control was run in the Cy3 channel for a baseline comparison. Ratios between the Cy5 and Cy3 channel were used in further analyses. Ratios were selected over intensities as they displayed a normal distribution about zero and accounted for potential array effects. Only experiments that respired (n = 47 conditions) were considered in this analysis. A full description of the design, materials, and methods was previously presented [13, 37], and the raw data is freely available at NCBI GEO under the accession number of GSE26288.

All transcripts and arrays were standardized to have a mean ratio of zero and a variance of one. The *j* ^*th*^ eigengene, *z* ^*j*^, is generated by performing a PCA on the *j* ^*th*^ cluster. The gene expression data *Y* ^*j*^ is defined for each set of gene transcripts identified with K-means clustering. A singular value decomposition (SVD) is performed on *Y* ^*j*^ as follows:

$$Y^{j}=U^{j}D^{j}{\left(V^{j}\right)}^{T},$$ (1)

with *U* ^*j*^ and *V* ^*j*^ orthogonal, *D* ^*j*^ diagonal, and *Y* ^*j*^ standardized matrices. To infer the membership of each cluster, we use K-means clustering with 1000 random initializations and 100 clusters. The SVD in equation (1) is used to extract the first eigengene for each cluster by taking the first eigenvector from the matrix $U_{\left(1\right)}^{j}=z^{j}$. These clusters (along with the corresponding environmental conditions) are presented in the supplemental materials (S2 File).

### Heatmap Analysis

The presented heatmap was constructed in R v.3.0.2 using the heatmap.2 function. We standardized both the eigengenes and experimental variables to have a mean of zero and a variance of one for visualization purposes. The colors vary from blue (negative log-ratios for transcripts or negative log values for variables) to red (positive log-ratios for transcripts or positive log values for variables). The K-means clusters of gene transcripts and experiments are organized in the y- and x-axes, respectively, based on hierarchal clustering. To allow easier visualization of the data, white spaces were included between groups of variables that had distances greater than 0.9 × (maximum hierarchical distance).

### SParse eIgengene NEtwork model

Similar to Logsdon et al. [17], we assume the gene expression data is generated conditional on experimental conditions based on a conditional Gaussian graphical model as follows:

$$z_{i}|x_{i}\overset{iid}{∼}N\left(\Gamma x_{i},\;\Theta_{zz}^{-1}\right)$$ (2)

for the *i* ^*th*^ sample of *n* independent and identically distributed (*iid*) observations. In this case, **z**_**i**_ is a vector of eigengene values for the *i* ^*th*^ sample, and **x**_**i**_ is a vector of the experimental condition values for the *i* ^*th*^ sample. The matrix $\text{Γ}\;={\text{Θ}}_{\text{zz}}^{\text{-1}}{\text{Θ}}_{\text{zx}}$ captures the effects of the experimental conditions on eigengenes, the inverse covariance matrix ${\text{Θ}}_{\text{zz}}^{\text{-1}}$ captures the conditional dependence and independence relationships among eigengenes, and the matrix **Θ**_**zx**_ captures the effects of the experimental conditions on eigengenes conditional on all eigengenes in the model. A network is then learned among the eigengenes by inferring the non-zero structure of ${\text{Θ}}_{\text{zz}}^{\text{-1}}$ and **Θ**_**zx**_ across the experimental conditions by using the following approach that combines the vbsr algorithms with the network reconstruction previously proposed in two publications [17, 22].

### Variational Bayes spike regression

We infer the neighborhoods (i.e., the non-zero elements of each row of **Θ**_**zz**_ and **Θ**_**zx**_) of each eigengene through a penalized regression approach [17]. We use the vbsr algorithm proposed in Logsdon et al. 2012 [22] in which a given eigengene is modeled as follows:

$$\begin{array}{c} z_{i}^{j}=\sum_{k\neq j}\beta_{k}^{z}z_{i}^{k}+\sum_{l}\beta_{l}^{x}x_{i}^{l}+e_{i} \end{array}$$ (3)

with the improper prior

$$\begin{array}{c} \beta_{j}∼p_{\beta }I\left[\beta_{j}\neq 0\right]+\left(1-p_{\beta }\right)I\left[\beta_{j}=0\right] \end{array}$$ (4)

where *β*_*j*_ indicates either a specific *β* ^*z*^ or *β* ^*x*^, *p*_*β*_ is the prior probability that any *β*_*j*_ is non-zero, I[*x*] is the indicator function, and *e*_*i*_ is an independently, identically, and normally distributed error term. We define a reparameterization of *p*_*β*_ as ℓ_0_ = 2 (log {*p*_*β*_} − log {1 − *p*_*β*_} − *π*). The output of the vbsr model are Z-statistics for eigengene effects to test the null hypotheses $H_{0}\;:\;\beta_{k}^{z}=0$ ($Z_{k}^{z}$) or experimental effects to test the null hypothesis $H_{0}\;:\;\beta_{l}^{x}=0$ ($Z_{l}^{x}$). If we reject a null hypothesis (e.g. $\left|Z_{l}^{x}\right|\;>\;Z_{crit})$, then we declare there is an edge between environmental condition l and eigengene j after a Bonferroni multiple testing correction (*Z*_*crit*_ = *P* ^−1^ (0.025/14200), for a standard normal random variable). Accordingly, this corresponds to the element $\theta_{zx}^{lj}$ of **Θ**_**zx**_ being non-zero. As in Logsdon et al. [17], for each eigengene-eigengene interaction, we consider the estimate from both directions of regression (e.g., eigengene k regressed on eigengene j, and vice versa) and aggregate the evidence. As opposed to averaging the posterior probabilities, we geometrically average the p-values from the Z-statistics for each direction of regression: ${\text{log}}_{10}\;\left(P_{jk}^{avg}\right)\;=\;{\text{log}}_{10}\;\left(P_{jk}\right)/2+{\text{log}}_{10}\;\left(P_{kj}\right)/2$, where $P_{jk}=P\left(\left|X\right|\;>\;\left|Z_{k}^{z}\right|\right)$ for a standard normal random variable *X*.

We select the penalty parameter $\ell_{0}=-F^{-1}\;\left(1-\;\frac{005}{m}\right)\;-\;log\left(n\right)\;+\;2log\left(1\;-\;p\right)\;-\;2log\left(p\right)$ with *p* = 0.95 and *F* ^−1^(*x*) as the inverse cumulative density function of a $\chi_{1}^{2}$ random variable. This penalty parameter selection prevents over-fitting and provides a more computationally efficient solution to the penalized regression problem than running the model on a grid of penalty parameters. The final network is determined based on a Bonferroni cutoff for a significance threshold of 0.05 (*α* = 0.05/14200 = 3.52 × 10^−6^) for the Z-statistics. Five-hundred random restarts are run for each regression, and approximate Bayesian model averaging is performed across all identified models as described in Logsdon et al. [22].

### Weighted Graph Correlation Network Analysis (WGCNA)

We performed a soft thresholded WGCNA [18, 19]. This analysis defines candidate correlation networks based on 𝒢^*η*^ = abs (cor (*Z*))^*η*^, where *η* = 1,…, 10 and *Z* = [*Y*, *X*] in which *Y* is the data matrix of 1,419 gene expression variables and *X* is the data matrix of 42 experimental condition variables. Additionally, cor(*Z*) is the correlation matrix of the variables in *Z*. The network with the most scale-free topology was selected (in this case *η* = 10), and clustering was performed using the distance metric defined by 1 − 𝒢^10^ with the partitioning around medoids algorithm and 100 clusters.

All of the code required to run these analyses is detailed in the supplemental materials (S3 File).

### Visualization of the network

The resulting network was visualized in the software package Cytoscape (v. 3.0, www.cytoscape.org). The network was organized with an unweighted, spring-embedded layout. The node size was scaled to be proportional to the number of transcripts in each cluster. Additionally, the nodes were colored based on key features: experimental conditions are colored yellow, predicted RDase-neighboring transcripts are colored red, and other non-RDase oxidoreductases putatively identified as involved in respiration are colored blue. The Cytoscape file is available in the supplementary materials (S4 File).

### Follow-up solvent toxicity experiments

#### Experimental setup

A culture containing *Dhc* strain 195 was grown under typical continuously-fed conditions in four 100-mL cultures as previously described [28, 32]. A syringe-pump (Cole-Parmer, Vernon Hills, IL) delivered a mixture of 1:4.4 PCE:butyrate on a molar basis at a rate of 2.4 µmol PCE/hour. After 24 hours of continuous feeding, two of the cultures received an injection of 1.37 mmol PCE/L (140 µL neat PCE/L media), well above the theoretical solubility of 905 µmol PCE/L (for a 160 mL vessel with 100 mL of media at 30°C). Gas chromatography (GC) samples were taken throughout the study to analyze substrates and products of respiration. For RNA samples, 2 mL liquid media samples were withdrawn at 0, 12, 24, 30, 36, 48, and 72 hours elapsed since the beginning of the experiment, and the cells were pelleted as previously described [28, 32].

#### GC methods

Chlorinated-ethenes and ethene were assayed utilizing a gas chromatography (GC) flame-ionization (FID) detection method as previously described [28, 32, 37].

#### Primer design

Primers for DET0137, the putative methylglyoxal synthase, and for DET0097, a putative iron-dependent regulator, were designed and ordered from Integrated DNA Technologies (IDT, Coralville, IW). In addition to these primers, the previously reported specific primers for the DET0110 *hup* hydrogenase and *Dhc* 16S rRNA were also used to monitor the expression of reference RNAs [32, 37]. The sequences for all primers are provided in S2 Table.

#### qRTPCR

RNA was extracted from the sample pellets using the Ambion RNEasy Mini Extraction Kit (Life Technologies, Grand Island, NY) as previously described [28, 32]. The RNA was quantified on a Nanodrop 2000c (Thermo Scientific, Wilmington, DE) and the quality was confirmed using a RNA 6000 Nano Kit on an Agilent Bioanalyzer (Agilent Technologies, Santa Clara, CA) according to the manufacturer’s protocols. The RNA was reverse transcribed into cDNA using the Cyscribe Kit (Bio-Rad, Hercules, CA) as previously described [28, 31, 32, 37]. The qRTPCR was run for both samples and long amplicon standards of known concentrations on a BioRad iCycler (Bio-Rad) using a SYBRGreen method as previously described [28, 32]. The results from the iCycler were quantified using DART-PCR version 1.0 algorithms (www.gene-quantification.de; [38]) to obtain transcript copy numbers, which were then transformed into copies per mL of culture extracted.

## Supporting Information

S1 Table Gene expression cluster membership, gene annotations, and an indication of which genes are annotated as RDase-associated or other respiration-linked oxidoreductase genes (based on Seshadri et al., 2005) for all transcripts monitored on the *Dhc* strain 195 specific microarray. (XLSX)

S2 Table Sequences, annealing temperatures, and amplicon lengths of the qRTPCR and long amplicon (LA) primers for DET0097, DET0110, DET0137, and the 16S rRNA gene. (XLSX)

S1 File Description of experimental conditions considered in the model. The first column provides the identifier used in SPINE, the second column provides a brief description of the values and units, and the remaining columns provide the actual data recorded for each experimental condition.
(XLSX)

S2 File File containing all processed gene-expression data along with the experimental condition variables for the 47 samples. These data were used as input to the R scripts in S3 File during the analysis.
(R) Click here for additional data file.

S3 File Script implemented to run the vbsr package in R. The overall work-flow includes the separation of experimental conditions from gene expression values, the clustering of the gene expression values using a K-means analysis, and the construction of SPINE using vbsr. The output of this analysis is a network file that can be viewed in any network viewer (e.g., Cytoscape).
(TSV)

S4 File Cytoscape file of the SPINE network generated by this study. Experimental perturbations are represented as yellow nodes and are a constant size. The size of the transcript cluster nodes is a function of the number of transcripts in that cluster, and the transcript clusters are colored as either white to red or white to blue nodes. The nodes are colored based on the proportion of their constituent gene transcripts that are RDase-related (with white and red indicating a lower and higher proportion, respectively) or the proportion of their constituent genes identified as other oxidoreductases putatively involved in electron transport (with white and blue indicating a lower and higher proportion, respectively). Dashed lines indicate a negative relationship; solid lines indicate a positive relationship.
(CYS)
